# Development and characterization of an *Sf-1*-Flp mouse model

**DOI:** 10.1172/jci.insight.190105

**Published:** 2025-03-04

**Authors:** Marco Galvan, Mina Fujitani, Samuel R. Heaselgrave, Shreya Thomas, Bandy Chen, Jenny J. Lee, Steven C. Wyler, Joel K. Elmquist, Teppei Fujikawa

**Affiliations:** 1 Center for Hypothalamic Research, Department of Internal Medicine,; 2 Department of Neuroscience,; 3 Department of Pharmacology, and; 4 Peter O’Donnell Jr. Brain Institute, UT Southwestern Medical Center, Dallas, Texas, USA.; 5 Institute of Human Life and Ecology, Osaka Metropolitan University, Osaka, Japan.

**Keywords:** Metabolism, Neuroscience, Diabetes, Glucose metabolism

## Abstract

The use of genetically engineered tools, including combinations of Cre-LoxP and Flp-FRT systems, enables the interrogation of complex biology. Steroidogenic factor-1 (SF-1) is expressed in the ventromedial hypothalamic nucleus (VMH). Development of genetic tools, such as mice expressing Flp recombinase (Flp) in SF-1 neurons (*Sf-1*-Flp), will be useful for future studies that unravel the complex physiology regulated by the VMH. Here, we developed and characterized *Sf-1*-Flp mice and demonstrated their utility. The Flp sequence was inserted into the *Sf-1* locus with P2A. This insertion did not affect *Sf-1* mRNA expression levels and *Sf-1*-Flp mice do not have any visible phenotypes. They are fertile and metabolically comparable to wild-type littermate mice. Optogenetic stimulation using adeno-associated virus (AAV) carrying Flp-dependent channelrhodopsin-2 (ChR2) increased blood glucose and skeletal muscle PGC-1α in *Sf-1*-Flp mice. This was similar to SF-1 neuronal activation using *Sf-1*-BAC-Cre and AAV carrying Cre-dependent ChR2. Finally, we generated *Sf-1*-Flp mice that lack β2-adrenergic receptors (*Adrb2*) only in skeletal muscle with a combination of Cre/LoxP technology (*Sf-1*-Flp:SKM^ΔAdrb2^). Optogenetic stimulation of SF-1 neurons failed to increase skeletal muscle PGC-1α in *Sf-1*-Flp:SKM^ΔAdrb2^ mice, suggesting that *Adrb2* in skeletal muscle is required for augmented skeletal muscle PGC-1α by SF-1 neuronal activation. Our data demonstrate that *Sf-1*-Flp mice are useful for interrogating complex physiology.

## Abstract 2

We generated a novel trasngenic animal, *Sf-1*-Flp mice. We functionally validated the model and demonstrated its utility for untangling complex physiology.

## Introduction

The ventromedial hypothalamic nucleus (VMH) regulates multiple physiological responses and has been investigated for decades (1–3). For instance, the VMH regulates metabolism, food intake, behavior such as defensive behavior, reproduction, autonomic nervous system, and many other physiological functions (1–4). Although the role of the VMH in various physiological functions is well investigated, the precise molecular and neuronal mechanisms by which the VMH regulates physiology remain largely unclear.

Steroidogenic factor-1 (SF-1, gene *Nr5a1*) is expressed in the entire VMH early in embryonic development (5–7). In adults, SF-1 expression becomes more restricted to VMH neurons in the dorsomedial and center of the VMH (5, 6). Of note, SF-1 is also expressed in the pituitary gland, adrenal gland, and gonads like the testis and ovary (8, 9). We previously demonstrated that SF-1 neurons in the VMH (VMH^SF-1^ neurons) regulate skeletal muscle transcriptional events via sympathoadrenal drive (10). Briefly, optogenetic stimulation of VMH^SF-1^ neurons using *Sf-1*-BAC-Cre (11) and adeno-associated virus (AAV) carrying Cre-dependent channelrhodopsin-2 (ChR2) (AAV-DIO-ChR2) (12) increases mRNA levels of skeletal muscle peroxisome proliferator-activated receptor γ coactivator 1-α (PGC-1α, gene *Ppargc1a*) (10). Surgical ablation of the adrenal gland or global knockout of β2-adrenergic receptors (*Adrb2*) hampers induction of skeletal muscle PGC1-α expression, suggesting the sympathoadrenal drives and *Adrb2* are required for VMH^SF-1^ neuronal regulation of skeletal muscle. Considering that *Adrb2* encodes a major subtype of adrenergic receptors in skeletal muscle cells (13), VMH^SF-1^ neurons regulate skeletal muscle PGC1-α likely via direct actions on *Adrb2* in skeletal muscle cells. However, *Adrb2* is expressed in other metabolically important tissues, including the pancreas (14), liver (15), and VMH (16); thus, it is still unclear which tissues are responsible for VMH^SF-1^ neuronal regulation of skeletal muscle PGC-1α expression when using global *Adrb2*-knockout mice. To determine whether *Adrb2* in skeletal muscle cells is indeed required for VMH^SF-1^ neurons to regulate skeletal muscle PGC-1α, we needed to develop an animal model that facilitates studies to ablate *Adrb2* only in skeletal muscle cells and simultaneously to manipulate VMH^SF-1^ neuronal activity. To this end, we developed mice expressing Flpe recombinase (Flp) in SF-1 neurons (*Sf-1*-Flp). Similar to the Cre-LoxP system, Flp recognizes FRT sites and induces a variety of recombinations depending on the direction and location of FRT sites (17–20). Because there is minimal overlapping enzymatic recognition between Cre-LoxP and Flp-FRT systems (21), these systems can be used in combination, enabling complex genetic manipulations.

Insertion of Flp into the *Sf-1* locus does not affect mRNA expression of *Sf-1* in the hypothalamus, pituitary gland, adrenal gland, and testis/ovary where SF-1 is expressed. We confirmed that *Sf-1*-Flp mice exhibit Flp activity in SF-1 neurons in the VMH without any visible phenotypes. Furthermore, fundamental metabolism such as body weight and glucose levels in *Sf-1*-Flp mice were comparable to littermate wild-type control mice. By crossing *Sf-1*-Flp with mice lacking *Adrb2* only in skeletal muscle by Cre-LoxP, we achieved activation of VMH^SF-1^ neurons in mice lacking *Adrb2* only in skeletal muscle. We found that lacking *Adrb2* only in skeletal muscle cells hampers the increase in skeletal muscle PGC-1α mRNA levels induced by VMH^SF-1^ neuronal activation. Collectively, our data demonstrate that the *Sf-1*-Flp mouse is a valuable tool to decipher physiology that is regulated by VMH^SF-1^ neurons.

## Results

### Generation of Sf-1-Flp mice.

**Figure 1:**
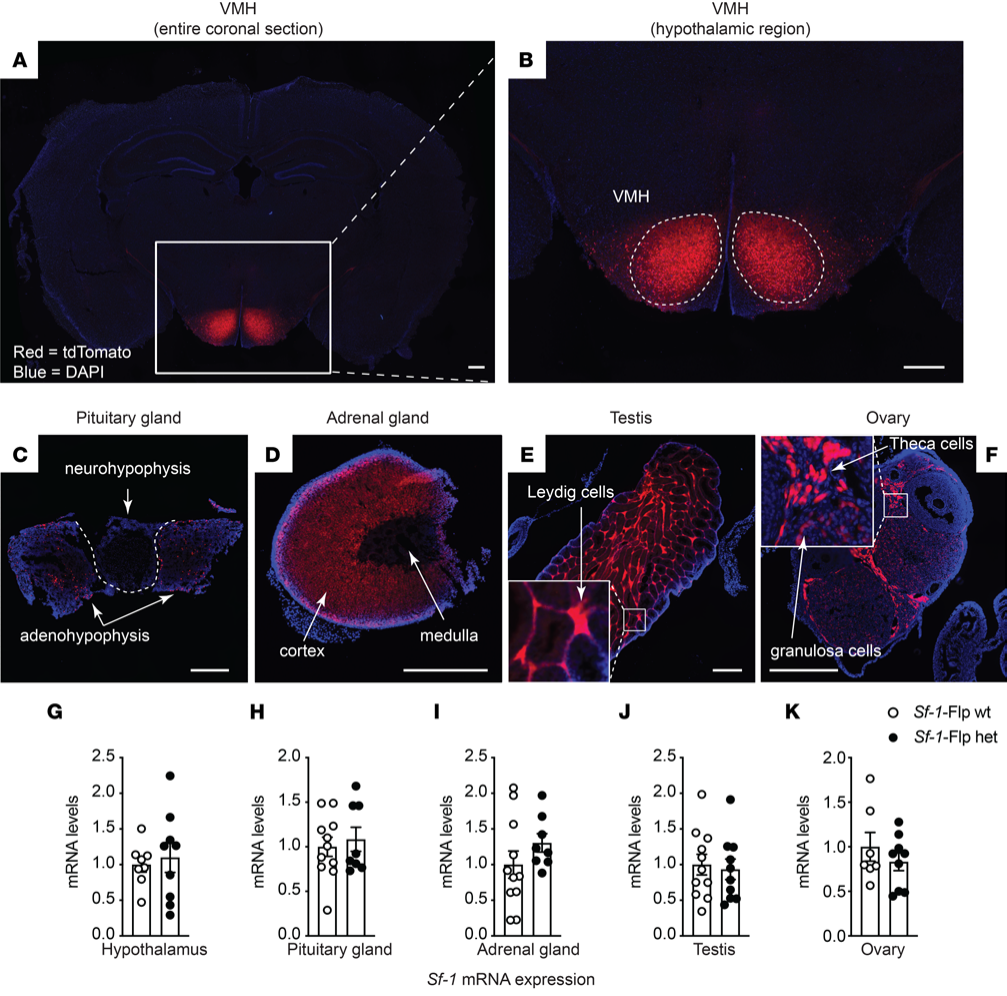
Generation of *Sf-1*-Flp mice. Representative images of (**A** and **B**) the ventromedial hypothalamic nucleus (VMH), (**C**) pituitary gland, (**D**) adrenal gland, (**E**) the testis, and (**F**) ovary of *Sf-1*-Flp:Ai65f mice. Ai65f mice bear Flp-dependent tdTomato in the *Rosa26* locus. Scale bars: 500 μm. mRNA levels of *Sf-1* in the (**G**) hypothalamus, (**H**) pituitary gland, (**I**) adrenal gland, (**J**) the testis in males, and (**K**) ovary in females of *Sf-1*-Flp heterozygous mice. The control group was composed of wild-type littermate mice. Values are mean ± SEM (*n* = 8–11). A 2-tailed, unpaired *t* test was used.

We first attempted to insert Flpe recombinase, a temperature-stabilized form of Flp (22), downstream of the *Sf-1* coding sequence using either IRES or P2A. However, we could not obtain any founders with either method. SF-1 global knockout is lethal in mice (23). We concluded that inserting Flp into the C-terminal region of SF-1 disrupted transcription of *Sf-1* by some unknown mechanisms, leading to the lethality. Thus, we decided to insert Flp upstream of the start codon of *Sf-1* with P2A, which is a self-cleaving peptide (24, 25) (Supplemental Figure 1; supplemental material available online with this article; undefinedDS1). Using the CRISPR/Cas9 approach as we described previously (26, 27), we successfully obtained *Sf-1*-Flp mice. We bred *Sf-1*-Flp mice with Ai65f^TB/TB^ mice, which bear Flp-dependent tdTomato in the *Rosa26* locus (*Sf-1*-Flp^+/–^:Ai65f^TB/–^) (28). *Sf-1*-Flp^+/–^:Ai65f^TB/–^ mice expressed tdTomato in tissues where SF-1 is expressed such as the VMH, pituitary gland, adrenal gland, and gonads (Figure 1, A–F). In the brain, tdTomato expression was restricted to the VMH and axons emanating from the VMH (Supplemental Figure 2). These expression patterns are consistent with previous reports (6, 29), suggesting no ectopic expression, at least in the brain. In line with previous studies (6, 11), a scattering of tdTomato^+^ cells was observed outside of the VMH (Figure 1, A and B, and Supplemental Figure 2). There are a couple of potential explanations for why these scattered tdTomato^+^ cells were observed outside of the VMH. Although this may simply be an artifact due to the nature of transgenic animals, the following explanation is more likely the case. During embryonic development, SF-1^+^cells migrate into the VMH from several areas such as the median eminence (6). Some of these SF-1^+^ cells during development fail to migrate into the VMH (6). Indeed, single-cell/single-nucleus RNA-sequencing (sc/sn-RNA-seq) studies have pointed out a small number of POMC^+^ cells, which are located in the hypothalamic arcuate nucleus (ARH), expressing *Sf-1* (30). Further studies using cutting-edge technology, such as spatial RNA-seq (31), will be warranted to determine genetic properties of these scattered cells outside of the VMH. Next, we determined whether the insertion of Flp disrupts expression of *Sf-1*. We found no differences in mRNA levels of *Sf-1* in the hypothalamus, pituitary gland, adrenal gland, and testis/ovary between heterozygous *Sf-1*-Flp mice and wild-type littermate control mice (Figure 1, G–K). These data suggest that the insertion of Flp into 5′-UTR of the *Sf-1* locus unlikely affects *Sf-1* mRNA expression in *Sf-1*-Flp mice.

### Sf-1-Flp mice have normal body weight and whole-body glucose metabolism.

**Figure 2:**
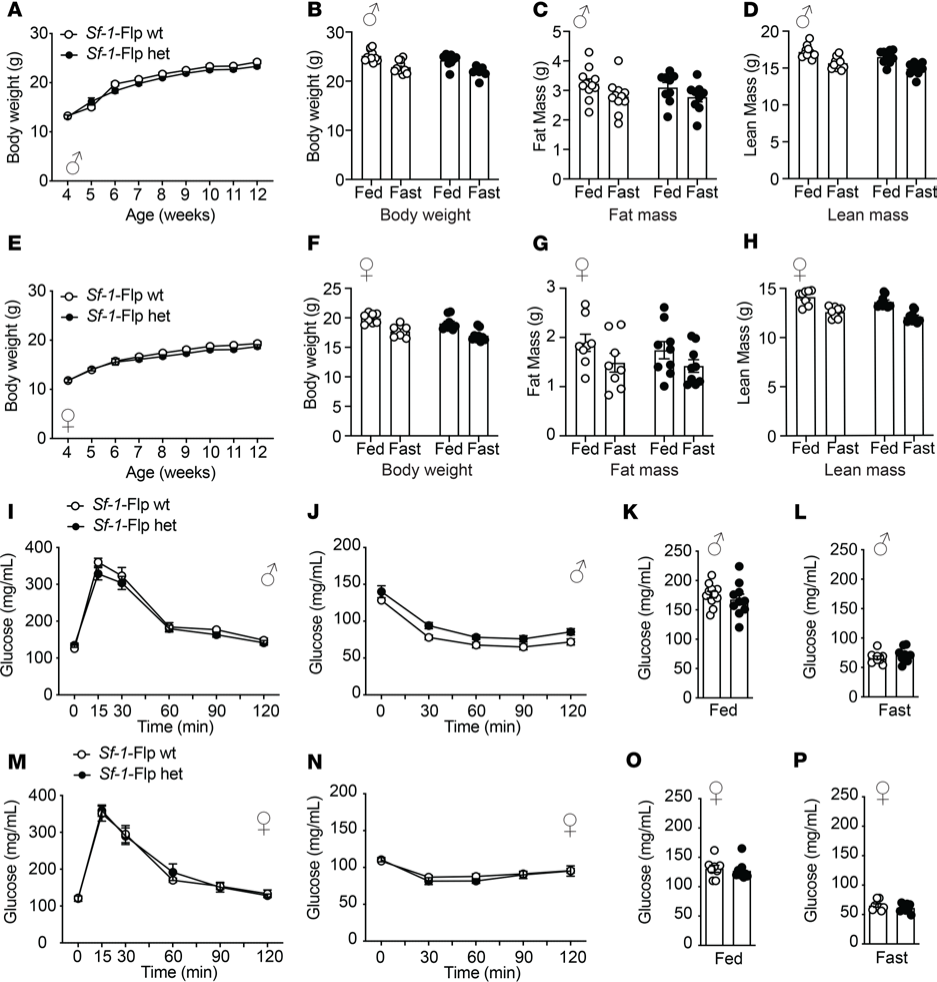
*Sf-1*-Flp mice do not have aberrant body weight and glucose metabolism. The time course of body weight of *Sf-1*-Flp heterozygous (**A**) male and (**E**) female mice. (**B** and **F**) Body weight, (**C** and **G**) fat mass, and (**D** and **H**) lean mass in fed and 24-hour fasted conditions in *Sf-1*-Flp heterozygous (**B**–**D**) male and (**F**–**H**) female mice. Glucose tolerance test in *Sf-1*-Flp heterozygous (**I**) male and (**M**) female mice. Insulin tolerance test in *Sf-1*-Flp heterozygous (**J**) male and (**N**) female mice. Blood glucose levels in fed and 24-hour fasted conditions in *Sf-1*-Flp heterozygous (**K** and **L**) male and (**O** and **P**) female mice. Values are mean ± SEM (*n* = 6–11). Detailed statical analysis is described in Supplemental Table 3. Briefly, 2-way repeated ANOVA was used in **A**, **E**, **I**, **J**, **M**, and **N**, 2-way ANOVA was used in **B**–**D** and **F**–**H**, and 2-tailed, unpaired *t* test was used in **K** and **L**. Bonferroni’s or Tukey’s multiple-comparison test was used for the ANOVA post hoc test.

**Figure 3:**
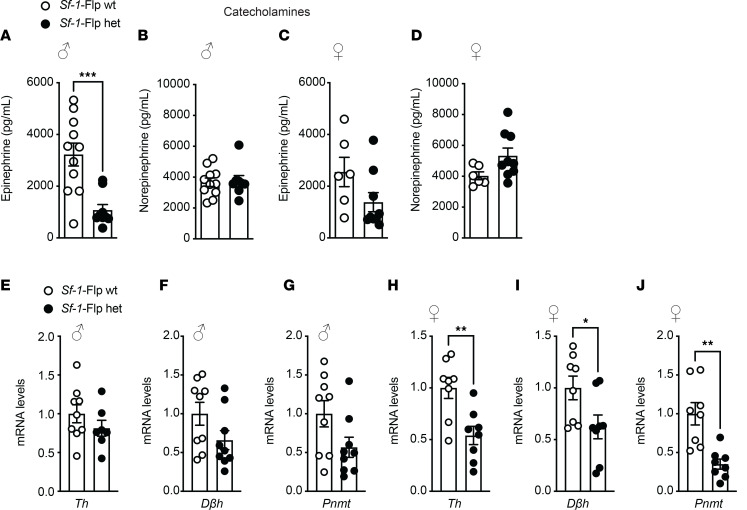
*Sf-1*-Flp mice have lower blood epinephrine. (**A**–**D**) Blood catecholamine levels in fed and 24-hour fasted conditions in *Sf-1*-Flp heterozygous male and female mice. (**B**–**J**) mRNA levels of tyrosine hydroxylase (*Th*), dopamine-β-hydroxylase (*Dbh*), and phenylethanolamine *N*-methyltransferase (*Pnmt*) in the adrenal gland in *Sf-1*-Flp heterozygous male and female mice. Values are mean ± SEM (*n* = 6–11). *P < 0.05, **P <0.01, ***P < 0.001 by 2-tailed, unpaired *t* test.

Next, we assessed baseline metabolic parameters in *Sf-1*-Flp mice by comparing heterozygous *Sf-1*-Flp mice with wild-type littermate control mice. Body weight, fat mass, and lean mass in *Sf-1*-Flp mice were comparable to those of control mice (Figure 2, A–H). There were no significant differences in glucose tolerance and insulin tolerance between control and *Sf-1*-Flp mice (Figure 2, I, J, M, and N). Furthermore, fed and fasted blood glucose levels were comparable in control and *Sf-1*-Flp mice (Figure 2, K, L, O, and P), suggesting that *Sf-1*-Flp mice do not have aberrant whole-body glucose metabolism. SF-1 is required for maintaining normal endocrine function in mice, as *Sf-1* heterozygous global knockout mice exhibit aberrant blood leptin and corticosterone levels (32). Blood leptin, insulin, and corticosterone levels in fed and fasting status were comparable in control and *Sf-1*-Flp mice (Supplemental Figure 3, A–C). We found that basal blood epinephrine levels in *Sf-1*-Flp male mice were significantly lower than in control mice (Figure 3A). In female mice, blood epinephrine levels tended to be lower than control mice, with the average value of epinephrine in *Sf-1*-Flp female mice being approximately 55% of the control group (Figure 3C). Blood norepinephrine levels were comparable between *Sf-1*-Flp and control mice (Figure 3, B and D), suggesting that the synthesis of epinephrine was disrupted. Thus, we investigated whether epinephrine synthesis in the adrenal gland was disrupted by measuring mRNA levels of tyrosine hydroxylase (*Th*), dopamine-β-hydroxylase (*Dbh*), and phenylethanolamine *N*-methyltransferase (*Pnmt*) in the adrenal gland, which are essential for epinephrine synthesis. mRNA levels of *Th*, *Dbh*, and *Pnmt* in male adrenal glands tended to be lower than control mice (Figure 3, B–G), and in females, they were significantly lower than control mice (Figure 3, H–J). These results suggested that the synthetic pathway of epinephrine is disrupted by an insertion of P2A-Flp into the 5′-UTR of the *Sf-1* locus. Overall, our data demonstrated that the insertion of P2A-Flp into the 5′-UTR of the *Sf-1* locus unlikely affects most SF-1 functions; however, it potentially has indirect effects on adrenal medulla functions.

### Optogenetic stimulation using Sf-1-Flp mice.

**Figure 4:**
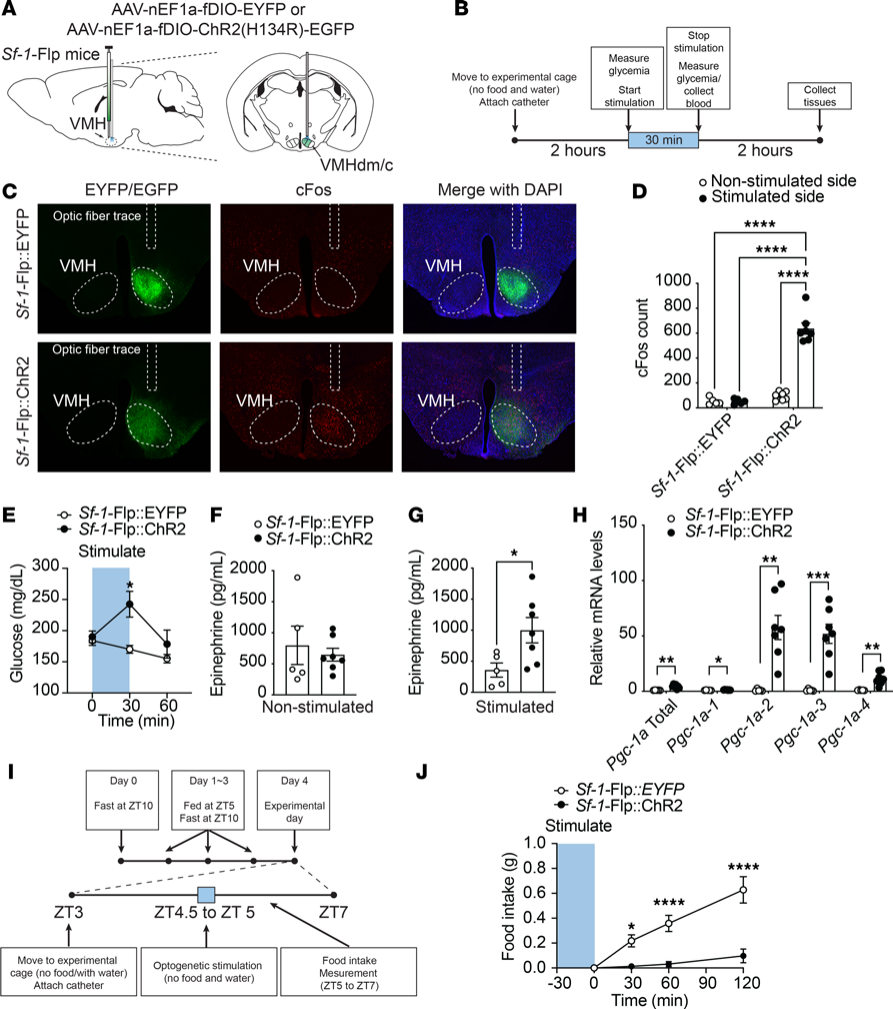
Optogenetic activation of VMH^SF-1^ neurons using *Sf-1*-Flp mice. (**A**) Schematic figure of targeting site of VMH^SF-1^ neurons using *Sf-1*-Flp mice and AAV containing Flp-dependent ChR2. (**B**) Experimental design and stimulus setting of optogenetics. (**C**) Representative figures of c-Fos expression pattern in the hypothalamus of mice expressing EGFP (*Sf-1*-Flp:EYFP) or ChR2 (*Sf-1*-Flp:ChR2) in VMH^SF-1^ neurons after optogenetic stimulation. (**D**) Number of c-Fos^+^ cells in the VMH of *Sf-1*-Flp:ChR2 mice after optogenetic stimulation. (**E**) Blood glucose levels in *Sf-1*-Flp:ChR2 mice with optogenetic stimulation. (**F** and **G**) Blood catecholamine levels and (**H**) mRNA expression levels of *Ppargc1a* isoform in skeletal muscle of *Sf-1*-Flp:ChR2 mice after optogenetic stimulation. (**I**) Experimental design for food intake study. (**J**) Food intake in *Sf-1*-Flp:ChR2 mice with optogenetic stimulation. All mice were male. Values are mean ± SEM (*n* = 5–7). *P < 0.05; **P <0.01; ***P < 0.001; ****P < 0.0001. Detailed statical analysis is described in Supplemental Table 3. Briefly, 2-way ANOVA was used in **D** and **J**, 2-way repeated-measures ANOVA was used in **E** and **J**, and 2-tailed, unpaired *t* test was used **E**–**H**. Bonferroni’s, Tukey’s, or Šidák’s multiple-comparison test was used for the ANOVA post hoc test.

Optogenetic stimulation of VMH^SF-1^ neurons using *Sf-1*-BAC-Cre mice and AAV-DIO-ChR2 increases blood glucose (10, 33) and mRNA levels of PGC-1α in skeletal muscle (10). Further, it suppresses appetite (34) and induces defensive behavior such as burst activities that include jumping and running (4, 35). To investigate whether we can utilize optogenetics in *Sf-1*-Flp mice, we delivered AAV bearing Flp-dependent ChR2 (AAV-fDIO-ChR2) into the VMH (*Sf-1*-Flp:ChR2) and examined whether optogenetic stimulation could phenocopy the stimulation in *Sf-1*-BAC-Cre mice. The control group received AAV that does not contain ChR2 but a fluorescent tag sequence (AAV-fDIO-EYFP) (*Sf-1*-Flp:EYFP). Optogenetic stimulation of VMH^SF-1^ neurons increased c-Fos in the VMH of *Sf-1*-Flp:ChR2 mice, suggesting that it is sufficient to induce augmented neuronal activities in VMH^SF-1^ neurons (Figure 4, C and D). As expected, optogenetic VMH^SF-1^ neuronal activation in *Sf-1*-Flp:ChR2 mice increased blood glucose (Figure 4E), and burst activities were observed. We confirmed that the stimulation of VMH^SF-1^ neurons significantly increased blood epinephrine (Figure 4, F and G) and mRNA levels of PGC1-α in tibial anterior (TA) skeletal muscle of *Sf-1*-Flp:ChR2 mice (Figure 4H). We further examined whether optogenetic stimulation of VMH^SF-1^ neurons in *Sf-1*-Flp:ChR2 mice suppresses appetite. Mice were fasted overnight, which maximized their appetite. The stimulation significantly reduced food intake even though they were fasted overnight (Figure 4, I and J). These results are a phenocopy of experiments using *Sf-1*-BAC-Cre and Cre-dependent AAV containing ChR2 (10). Overall, these data indicate that optogenetic tools can be used in *Sf-1*-Flp mice.

### Combination of Cre-LoxP and Flp-FRT system in Sf-1-Flp mice.

**Figure 5:**
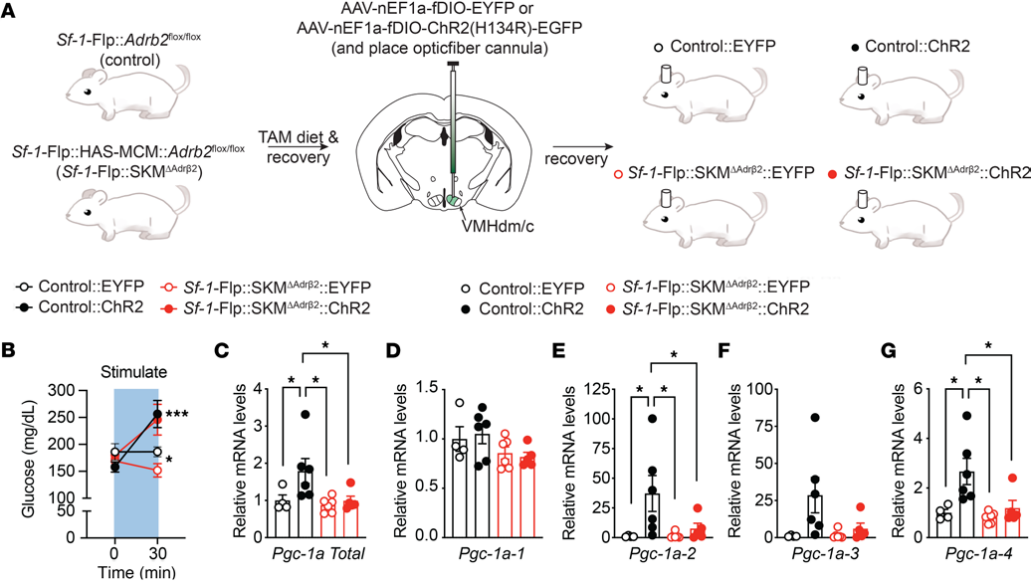
*Adrb2* in skeletal muscle cells is required for augmented skeletal muscle *Ppargc1a* mRNA by VMH^SF-1^ neuronal activation. (**A**) Experimental design to generate mice lacking *Adrb2* in skeletal muscle cells (*Sf-1*-Flp:SKM^ΔAdrb2^) and expressing ChR2 in VMH^SF-1^ neurons (*Sf-1*-Flp:SKM^ΔAdrb2^:ChR2). AAV-fDIO-EYFP was used as control AAV for AAV-fDIO-ChR2 (*Sf-1*-Flp:SKM^ΔAdrb2^:EYFP). A control group that does not lack *Adrb2* in skeletal muscle cells (control) was also administered either AAV-fDIO-EYFP or AAV-fDIO-ChR2 (Control:EYFP or Control:ChR2). (**B**) Blood glucose levels of *Sf-1*-Flp:SKM^ΔAdrb2^:ChR2 mice after optogenetic stimulation. (**C**–**H**) mRNA expression levels of *Ppargc1a* isoform in skeletal muscle of *Sf-1*-Flp:SKM^ΔAdrb2^:ChR2 mice after optogenetic stimulation. All mice were male. Values are mean ± SEM (*n* = 4–6). *P < 0.05, ***P < 0.001. Detailed statical analysis is described in Supplemental Table 3. Briefly, 2-way repeated-measures ANOVA was used in **B**, and 2-way ANOVA was used in **C**–**E**, **G**, and **H**. Šidák’s multiple-comparison test or the 2-stage linear step-up procedure of Benjamini, Krieger, and Yekutieli was used for the post hoc test.

Finally, we tested whether *Adrb2* in skeletal muscle cells is required for the induction of skeletal muscle PGC1-α mRNA by VMH^SF-1^ neuronal activation. To do so, we crossed *Sf-1*-Flp with mice expressing tamoxifen-inducible Cre under the control of the human skeletal muscle cell promoter (HAS-MCM) (36) and *Adrb2*-floxed (37) mice (*Sf-1*-Flp:SKM^ΔAdrb2^). As previous studies showed (38), we confirmed that Cre activity was induced in skeletal muscle of HAC-MCM mice by tamoxifen-containing (TAM) diet (Supplemental Figure 4A). The control group was composed of littermate *Sf-1*-Flp^+/–^:*Adrb2^fl/fl^* mice (control). All mice were fed a TAM diet. After recovery from the TAM diet, AAV-fDIO-EYFP or AAV-fDIO-ChR2 was microinjected into the VMH of control or *Sf-1*-Flp:SKM^ΔAdrb2^ mice (Control:EYFP, Control:ChR2, *Sf-1*-Flp:SKM^ΔAdrb2^:EYFP, and *Sf-1*-Flp:SKM^ΔAdrb2^:ChR2) (Figure 5A). Deletion of *Adrb2* in skeletal muscle cells did not affect the increase in blood glucose levels induced by VMH^SF-1^ neuronal activation (Figure 5B). In line with Figure 4, optogenetic VMH^SF-1^ neuronal activation in Control:ChR2 mice significantly increased skeletal muscle PGC1-α mRNA compared with Control:EYFP (Figure 5, C–H), whereas deletion of *Adrb2* in skeletal muscle cells significantly hampered the augmented skeletal muscle PGC1-α mRNA levels induced by VMH^SF-1^ neuronal activation (Figure 5, C–H). Of note, the TAM diet significantly reduced mRNA levels of *Adrb2* in skeletal muscle of *Sf-1*-Flp:SKM^ΔAdrb2^, but not that of control mice (Supplemental Figure 4B). These data demonstrate that *Adrb2* in skeletal muscle cells is required for the increase in skeletal muscle PGC1-α mRNA induced by VMH^SF-1^ neuronal activation. In addition, these results show that *Sf-1*-Flp mice can be used with combinations of the Cre-LoxP system.

## Discussion

In this study, we generated *Sf-1*-Flp mice that express Flpe recombinase in SF-1 cells and demonstrate that *Sf-1*-Flp mice can be useful in investigating complex physiology. Our previous report indicates that *Adrb2* contributes to VMH^SF-1^ neuronal regulation of skeletal muscle transcriptional events (10). However, it was unclear whether VMH^SF-1^ neurons regulate a direct action of *Adrb2* in skeletal muscle cells because *Adrb2* is widely expressed throughout the body in both rodents and humans (39–41). Our current study demonstrates that *Adrb2* in skeletal muscle cells is indeed required for the augmented skeletal muscle PGC1-α mRNA induced by VMH^SF-1^ neuronal activation. This strongly suggests that a direct action of *Adrb2* in skeletal muscle cells is key for VMH^SF-1^ neuronal regulation of skeletal muscle transcriptional events and functions.

VMH^SF-1^ neurons contribute to beneficial effects of exercise, including reductions in fat mass (42). Previous studies indicate that VMH^SF-1^ neurons are key to exercise-induced skeletal muscle adaptations via the sympathetic nervous system (10, 42). Our data (Figure 5) pinpoint that *Adrb2* in skeletal muscle cells is likely required for VMH^SF-1^ neurons to regulate skeletal muscle function, although further studies are needed to elucidate what precise physiological functions VMH^SF-1^ neurons contribute to regulation in the context of long-term exercise training. These functions may include the mitochondrial respiratory system and switching fiber-type composition. For instance, long-term exercise training has been shown to improve mitochondrial function and alter muscle fiber type (43–45). If VMH^SF-1^ neuronal activation is repeated for a certain duration (e.g., 8 to 12 weeks), then it may enhance mitochondrial function and influence fiber-type components, as recurrent exercise training does.

There are a couple of technical notes to be aware of for future studies when *Sf-1*-Flp mice are used. First, although we examined Flp activity in *Sf-1*-Flp mice throughout the brain (Supplemental Figure 2), we did not survey all peripheral tissues. Given the nature of genetically engineered mice, it is still possible that *Sf-1*-Flp mice may have ectopic Flp expression in some tissues where endogenous SF-1 is not expressed. Furthermore, we used tdTomato expression as a surrogate of Flp activity, but this does not guarantee that Flp works with other genetically engineered tools such as transgenic mice or AAV that bear FRT sites. Thus, it is important to validate each experimental model. Second, we found that *Sf-1*-Flp mice have lower blood epinephrine levels (Figure 3). Because *Sf-1* mRNA levels in the adrenal gland were comparable between *Sf-1*-Flp and control mice (Figure 1) and *Sf-1* is only expressed in the cortex of the adrenal gland, not the medulla where catecholamines are produced, it is unclear how the insertion of P2A-Flp into the 5′-UTR disrupts the production of epinephrine in the adrenal gland. Optogenetic studies show that *Sf-1*-Flp mice can increase blood epinephrine levels by VMH^SF-1^ neuronal activation, suggesting that *Sf-1*-Flp mice can modify epinephrine releases upon afferent neuronal inputs. Nonetheless, we suggest that it is ideal for studies to contain a control *Sf-1*-Flp group for proper interpretation of the results. Further investigations will be warranted on the mechanism underlying the effects of the transgenic insertion into the 5′-UTR of the *Sf-1* locus on blood epinephrine levels in *Sf-1*-Flp mice. Third, we noticed when AAV-fDIO-ChR2 was administered into the VMH of *Sf-1*-Flp mice, it took at least 5 to 6 weeks to have a metabolic phenotype induced by VMH^SF-1^ neuronal activation (e.g., increased glucose levels). When we use *Sf-1*-BAC-Cre mice and AAV-DIO-ChR2 (10), it only takes 2 to 3 weeks to observe a metabolic phenotype. The reason for these discrepancies in duration is unclear. However, it is important to keep this in mind when AAV approaches are used in *Sf-1*-Flp mice.

*Sf-1*-Flp mice will provide unique opportunities for those who investigate the physiology of SF-1^+^ cells, especially SF-1^+^ neurons in the VMH. As shown in Figure 5, *Sf-1*-Flp mice unlock the possibilities of conducting experiments that manipulate genes in peripheral cells and modulate SF-1^+^ neuronal activity or their function simultaneously. Furthermore, *Sf-1*-Flp mice can be used for temporal conditioned manipulation combined with AAV approaches such as using AAV carrying Flp-dependent Cre recombinase (AAV-fDIO-Cre). For instance, leptin receptors (LEPR) in the VMH appear to be important in regulating metabolism (11, 46), but it may be intriguing to determine the role of LEPR in the VMH in adults. If AAV-fDIO-Cre is administered into the VMH of *Sf-1*-Flp mice bearing 2 LoxP sites in the LEPR locus (47) (*Sf-1*-Flp:*Lepr^fl/fl^* mice), we may be able to achieve knockdown of LEPR specifically in VMH^SF-1^ neurons in adults. These approaches enable circumventing potential developmental compensations that happen when conventional Cre lines are used (48) and may unravel the role of interested genes in SF-1 neurons in adults.

In addition, *Sf-1*-Flp mice will enable the investigation of the detailed function of subpopulation of SF-1^+^ neurons. Studies using sc/sn-RNAseq have revealed genetically distinct subpopulations of the VMH, suggesting that the VMH is an extremely heterogeneous nucleus (16, 49). To interrogate which neuronal subpopulations of the VMH contribute to different physiological functions, *Sf-1*-Flp mice can be valuable because they can be applied in studies such as those employing the INTERSECT method (21). For instance, LEPR is widely expressed in the hypothalamus, including the ARH or dorsomedial hypothalamic nucleus (DMH) (50, 51). The ARH and DMH are juxtaposed to the VMH; thus, it is technically difficult to target the LEPR only in the VMH by microinjections or other injection methods. Using the INTERSECT method in mice expressing Flp in SF-1^+^ neurons and Cre in LEPR-expressing neurons by crossing *Sf-1*-Flp and *Lepr*-IRES-Cre mice (52), targeting LEPR neurons only in the VMH can be achieved.

In conclusion, our data demonstrate that *Sf-1*-Flp mice can be a great asset for neuroscience, metabolism, and many other fields that investigate the roles of cells expressing SF-1.

## Methods

### Sex as a biological variable.

Both male and female mice were used in Figures 1–3. Male mice were used in optogenetic studies (Figures 4 and 5). We did not consider sex as a biological variable and the findings are equally relevant to both sexes, as our preliminary experiments (data not shown) demonstrated that both male and female mice showed increased in blood glucose and PGC1-α mRNA levels in skeletal muscle after the stimulation of VMH^SF-1^ neurons.

### Generation of Sf-1-Flp mice.

We utilized the CRISPR/Cas9 approach to generate *Sf-1*-Flp mice. The sequence of *Mus musculus Nr5a1* (ENSMUST00000028084.5) was obtained from the Ensembl Genome Database (53, 54). Exon 2 of the *Nr5a1* gene encoding the translational start sight was targeted using the following guide RNA: 5′-GUACGAAUAGUCCAUGCCCGGUUUUAGAGCUAUGCU-3′. For the HDR repair template, a 1499-bp single-stranded DNA Megamer (Integrated DNA Technologies) was used encoding the Flpe recombinase sequence immediately followed by the P2A self-cleaving peptide sequence (22, 25). The 5′ and 3′ homology arms were 45 and 92 bp, respectively. Guide RNA, trcRNA, Cas9 protein, and HDR Template (all from Integrated DNA Technologies) were administered through a pronuclear injection in C57BL/6NCrl (Charles River) zygotes by the UT Southwestern (UTSW) Transgenic Technology Center. A schematic design of the *Sf-1*-Flp insertion is described in Supplemental Figure 1. Founders were screened by PCR followed by Sanger sequencing. To validate Flp activity in situ, we bred founders with Ai65f mice that express Flp-dependent tdTomato in the *Rosa26* locus (28).

### Genetically engineered mice and mouse husbandry.

*Sf-1*-Flp mice were generated as outlined above. HAS-MCM (stock 025750) (36), Ai65f (stock 032864) (28), and Ai14 (stock 007914) (55) mice were purchased from The Jackson Laboratory. *Abrb2^fl/fl^* mice (15) were obtained from Florent Elefteriou at Baylor College of Medicine (Houston, Texas, USA), with the permission of Gerard Karsenty at Columbia University (New York, New York, USA). Ear or tail gDNA was collected from each mouse to determine its genotype. A KAPA mouse genotype kit (Roche) was used for PCR genotyping. The sequences of genotyping primers and expected band sizes for each allele are described in Supplemental Table 1. Mice were housed at room temperature (22°C–24°C) with a 12-hour light/dark cycle (lights on at 6 am, or 7 am during daylight saving time) and fed a normal chow diet (2016 Teklad global 16% protein rodent diets, Envigo). A magnetic resonance whole-body composition analyzer (EchoMRI) was used to analyze the fat and lean mass of mice. Mice were maintained in groups and singly housed after AAV injections and optic fiber probe insertions.

### AAV injections and optic fiber probe insertions.

rAAV5-nEF1α-fDIO-hChR2(H134R)-EGFP-WPRE-hGH polyA (5.5 × 10^12^ VM/mL; catalog, PT-1384) and rAAV-nEF1α-fDIO-EYFP-WPRE-hGH polyA (2.8 × 10^12^ VM/mL; catalog, BHV12400246) were purchased from Biohippo. AAVs were unilaterally administered into the right side of the VMH of mice using a UMP3 UltraMicroPump (WPI) with 10 μL NanoFil microsyringe (WPI) and NanoFil needle (WPI, NF33BV). The volume of AAVs was 500 nL at the rate 100 nL per minute, and the needle was left for another 5 minutes after the injection was finished. The coordinates of VMH microinjection were AP, −1.4; L, +0.5; and D, −5.5 (from bregma). The optic fiber probe was inserted at the following coordinates: AP, −1.4; L, +0.5; and D, −5.0. The configuration of the probe was 200 μm Core, 0.39 NA, Ø 2.5 mm ceramic ferrule, and 6 mm length (RWD Life science Inc). The fiber probe was secured by adhesion bond (Loctite 454). Mice were allowed to recover for at least 5 to 6 weeks after the AAV injections to fully express recombinant proteins.

### Optogenetics.

As we previously reported (10), a laser unit (Opto Engine LLC, MDL-III-470) was used for VMH^SF-1^ neuronal stimulations. The power of tips was set to approximately 1 mW/mm^2^. The customized transistor-transistor logic generator was built based on the design by the University of Colorado Optogenetics and Neural Engineering Core. A rotary joint patch cable (Thorlabs, RJPSF4 for LED and RJPFF4 for the laser) was used to connect to the laser unit. The quick-release interconnect (Thorlabs, ADAF2) was used to connect the rotary joint patch cable to the fiber probe attached to the mouse head. The stimulation cycle was set to 5 ms duration, 20 Hz, 2-second activation, and 2-second rest cycle. As shown in Figure 4B, the entire stimulation time was set to 30 minutes.

### Assessment of food intake.

Mice were singly housed for food intake measurement. As shown in Figure 3H, mice were acclimated to time-restricted food availability for 3 days. Water was available throughout acclimation and experimental days except for when 30 minutes of optogenetic stimulation was executed. Food was available from ZT5 to ZT10. On the experimental day, mice were moved to the experimental cages without food. After optogenetic stimulation was finished at ZT5, food and water were provided. Food weight was measured at 30, 60, and 120 minutes.

### Immunohistochemistry and c-Fos quantification.

Mouse brains were prepared as previously described (10, 56). Anti–c-Fos (Abcam, ab190289, lot GR3418522-1) and secondary fluorescent antibodies (Thermo Fisher Scientific, A-21203, lot WD319534) were used. Dilution factors for antibodies were 1:2000 for the primary and 1:200 for the secondary. Images were captured by a fluorescence microscopy (Leica DM6 B) or a slide scanner (Axioscan 7, Zeiss). Quantification of c-Fos^+^ cells was described previously (10). Briefly, the exposure of captured images was adjusted and the VMH region was clipped by Adobe Photoshop based on the mouse brain atlas (28). Clipped images were exported to Fiji (version 2.14 from https://imagej.net/software/fiji/), and the number of cells expressing c-Fos was counted by particle measurement function.

### Assessment of glucose, catecholamines, and hormone levels in the blood.

Blood glucose was measured by a glucose meter as previously described (42, 57, 58). Blood for leptin, insulin, and corticosterone measurement was collected by tail-nicking with a heparin-coated capillary glass tube. We removed food 2 hours before blood collection to avoid immediate postprandial effects on blood metabolites and hormones and defined this condition as fed status in this study. For catecholamine measurement, trunk blood (Figure 3A) or submandibular blood (Figure 4F) was used. Plasma catecholamine and hormone levels were measured as previously described (42, 58). Briefly, the plasma catecholamines were analyzed by the Vanderbilt Hormone Assay & Analytical Services Core. Plasma leptin (Crystal Chem Inc, 90030), insulin (Crystal Chem Inc, 90080), and corticosterone (Cayman Chemical, 501320) levels were determined by commercially available ELISA kits.

### Glucose and insulin tolerance tests.

For glucose tolerance tests, mice were fasted for 6 hours prior to testing starting at ZT2, with water provided ad libitum. For the glucose tolerance test, mice received an i.p. injection of glucose solution (2 g/kg per body weight; Thermo Fisher Scientific, A2494001). For insulin tolerance tests, mice were fasted for 4 hours prior to testing starting at ZT4, with water provided ad libitum. Mice received i.p. insulin (Humalin R, Eli Lilly) at 0.75 U/kg body weight for male mice and 0.50 U/kg body weight for female mice. Blood glucose was measured using a handheld glucometer (Bayer’s Contour Blood Glucose Monitoring System).

### Assessment of mRNA.

mRNA levels in tissues were determined as previously described (42, 59). The sequences of primers for qPCR using SYBR or the catalog of TaqMan probes were described previously (60) and are in Supplemental Table 2.

### Statistics.

The data are represented as mean ± SEM. GraphPad Prism version 10 was used for the statistical analyses and a *P* value of less than 0.05 was considered significant. A detailed analysis of each figure is described in Supplemental Table 3. The sample size was decided based on previous publications (26, 42, 56–59, 61–63), and no power analysis was used. Figures were generated using Prism version 10, Adobe Illustrator 2023 and 2025, and Adobe Photoshop 2023 and 2025.

### Study approval.

All procedures using mice were approved by the Institutional Animal Care and Use Committee of UTSW, and animal experiments were performed at UTSW. Animal care followed the NIH *Guide for the Care and Use of Laboratory Animals* (National Academies Press, 2011).

### Data availability.

All datasets generated during the current study are provided in the Supporting Data Values file. *Sf-1*-Flp mice are publicly available upon material transfer agreement between the UTSW Medical Center and the recipient institute.

## Author contributions

MG and MF designed, performed, and analyzed experiments, and edited the manuscript. MG and MF equally contributed to this study and are co–first authors; their order in the author list was decided based on the alphabetical order of their first names. They have the right to be listed as the first author on their resumes. SRH, ST, BC, and JJL performed experiments. SCW designed *Sf-1*-Flp materials such as gRNA and edited the manuscript. JKE supervised the study and edited the manuscript. TF designed the study, performed experiments, supervised the study, analyzed experiments, and wrote and finalized the manuscript.

## Supplementary Material

Supplemental data

Supplemental table 1

Supplemental table 2

Supplemental table 3

Supporting data values

## Version 1 03/04/2025

In-Press Preview

## Version 2 04/22/2025

Electronic publication
