# Supplementary material for: Development and characterization of an Sf-1-Flp mouse model
Source: JCI Insight. 2025 Mar 4;10(8):e190105. doi: 10.1172/jci.insight.190105 (PMC12016925; doi:10.1172/jci.insight.190105)
Supplement: Supplemental data [file jciinsight-10-190105-s216.pdf]

SFigure 1

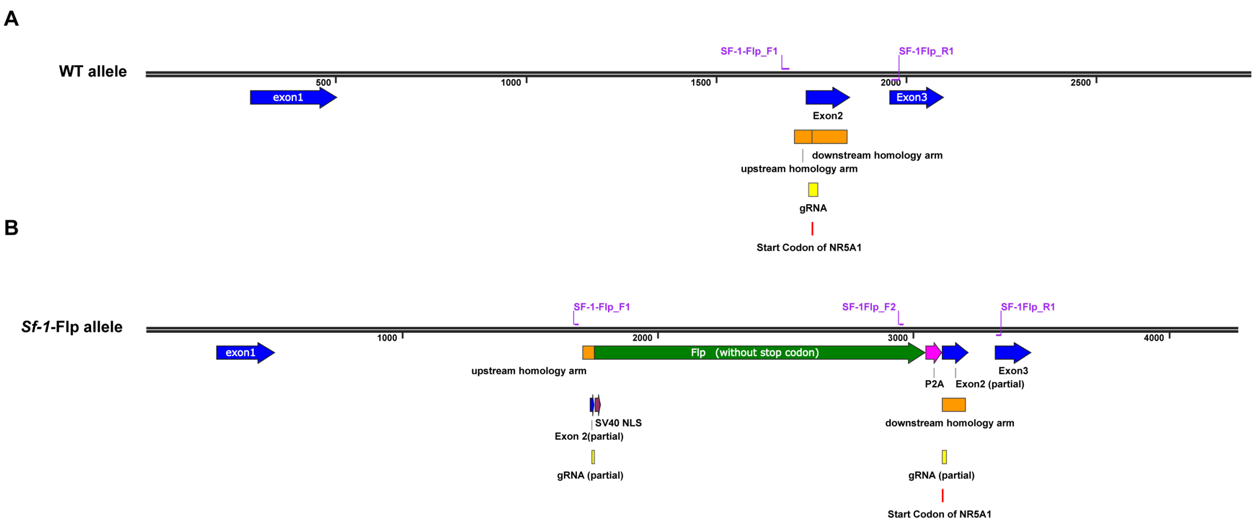

Supplemental Figure 1. The schematic design of *Sf-1*-Flp mice. WT allele (upper) and *Sf-1*-Flp allele (lower) sequences are described. SF-1-Flp_F1, F2, and R3 indicate the location of genotype primers for *Sf-1*-Flp mice.

SFigure 2

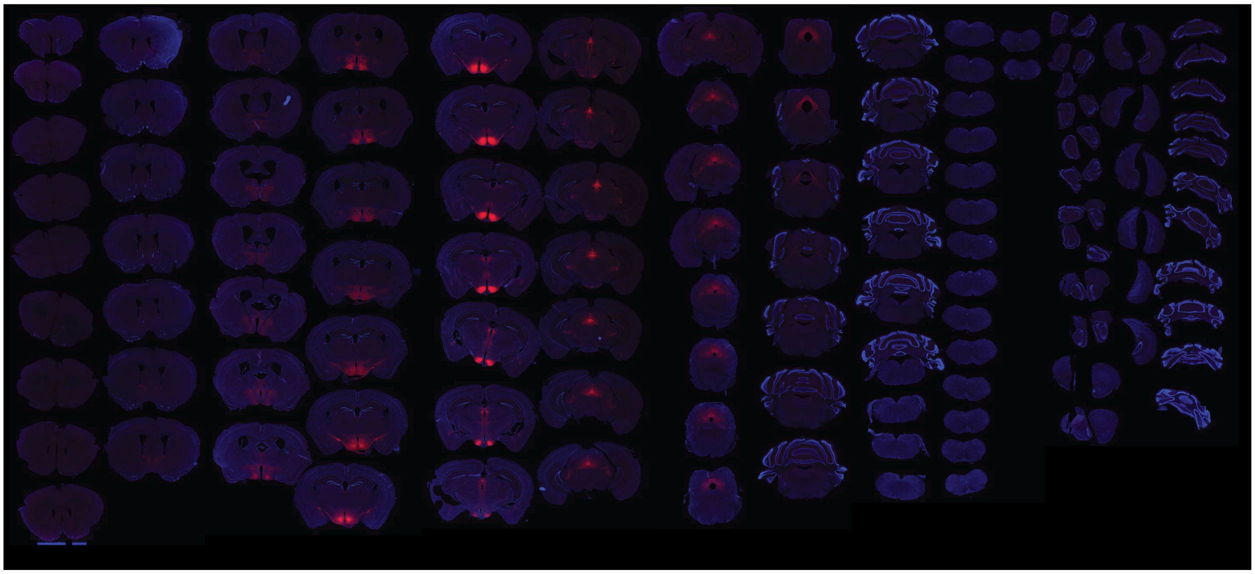

Supplemental Figure 2. Brains sections of *Sf-1*-Flp::Ai65f$^{TB/-}$ mice. Blue color indicates DAPI staining and red color indicates SF-1 positive cells.

SFigure 3

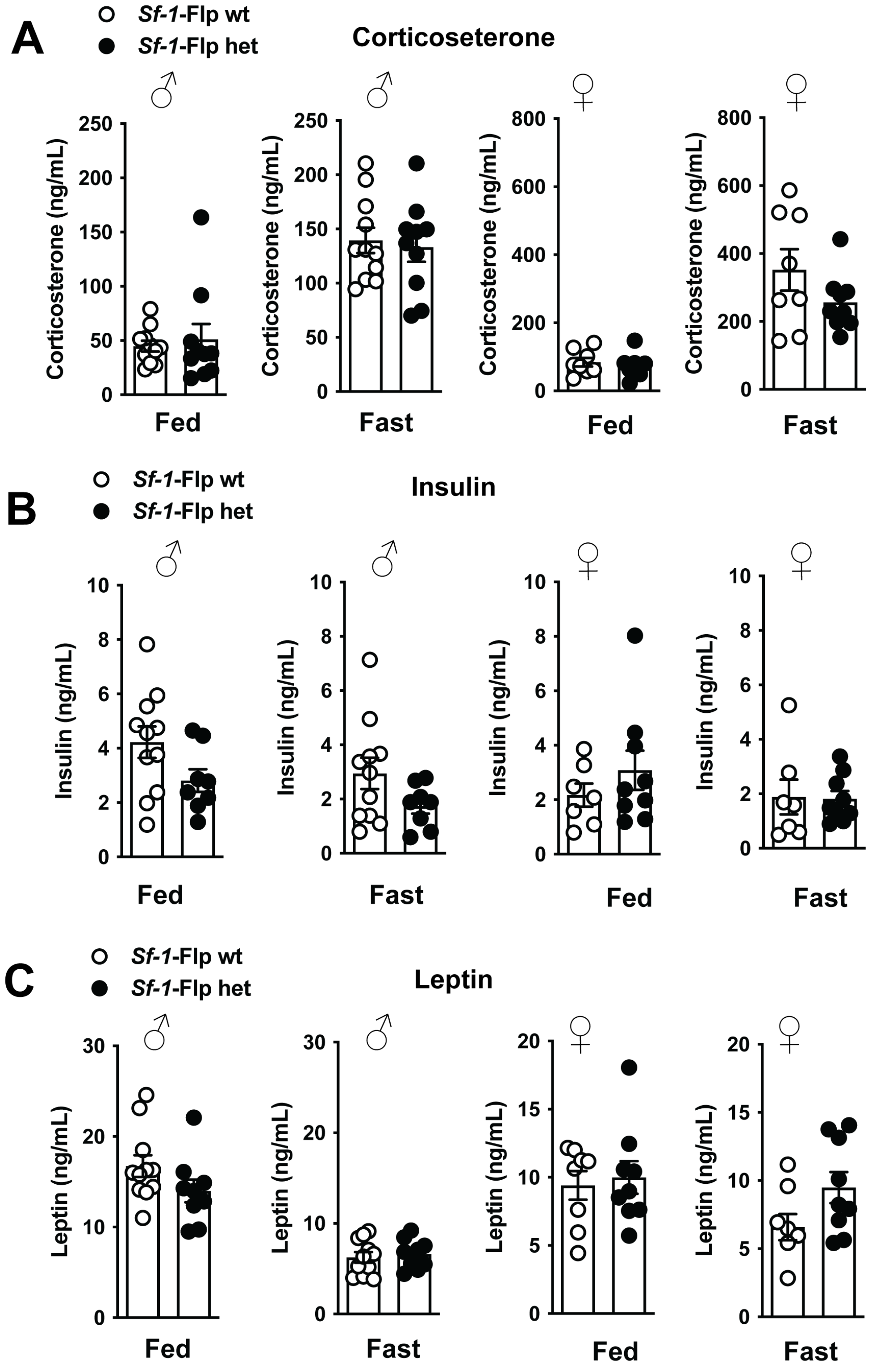

A
Sf-1-Flp wt
Sf-1-Flp het
Corticoseterone
♂
♀
Corticosterone (ng/mL)
0
50
100
150
200
250
200
400
600
800
Fed
Fast
B
Sf-1-Flp wt
Sf-1-Flp het
Insulin
Insulin (ng/mL)
0
2
4
6
8
10
Fed
Fast
C
Sf-1-Flp wt
Sf-1-Flp het
Leptin
Leptin (ng/mL)
0
10
20
30
5
15
Fed
Fast

Supplemental Figure 3. *Sf-1*-Flp mice have normal responses of corticosterone, leptin, and insulin to fasting challenge. Blood (**A**) corticosterone, (**B**) insulin, (**C**) and leptin levels in fed and 24-hour fast conditions in *Sf-1*-Flp heterozygous male and female mice. Values are mean ± S.E.M. Two-tailed unpaired t test was used. N = 7-11.

SFigure 4

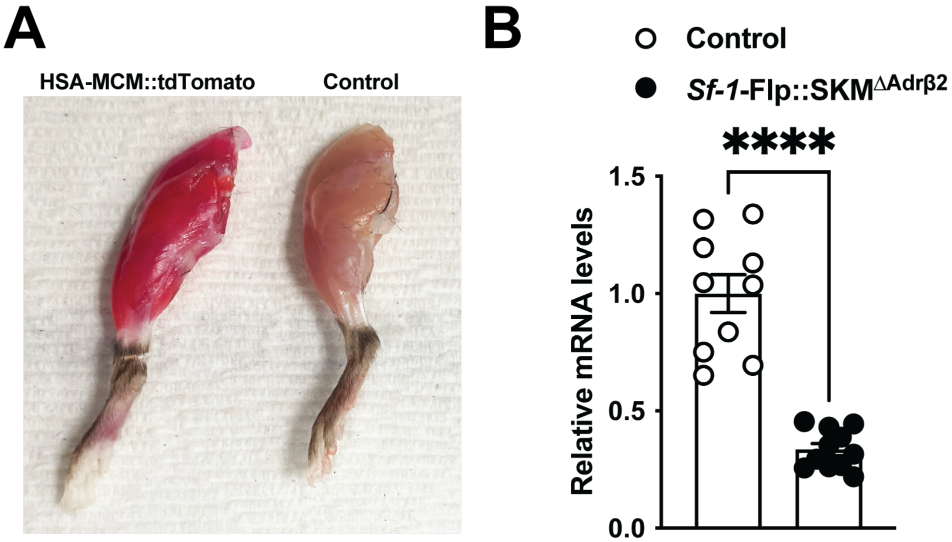

Supplemental Figure 4. *Sf-1*-Flp::SKM$^{\Delta Adr\beta 2}$ mice has lower Adrb2 mRNA expression in skeletal muscle. (**A**) Representative figure of lower limbs of HAC-MCM::Ai14$^{TB/-}$ mice after 4 weeks tamoxifen (TAM) diet. A control group is composed of Ai14 mice. All mice were fed with 4 weeks TAM diet. (**B**) mRNA levels of *Adrb2* in TA skeletal muscle in *Sf-1*-Flp::SKM$^{\Delta Adr\beta 2}$ mice after 4 weeks tamoxifen diet. A control group is composed of *Sf-1*-Flp$^{+/-}$::*Adrβ2*$^{flox/flox}$ mice. All mice were fed with 4 weeks TAM diet. Values are mean ± S.E.M. **** $p < 0.0001$. Two-tailed unpaired t test was used. N = 8-11
